# Grain Boundary Guided Folding of Graphene for Twisted Bilayer Graphene

**DOI:** 10.3390/nano15070482

**Published:** 2025-03-24

**Authors:** Feiru Feng, Kun Zhou, Kang Zhang, Liya Wang, Ruijie Wang, Jun Xia, Chun Tang

**Affiliations:** Faculty of Civil Engineering and Mechanics, Jiangsu University, Zhenjiang 212013, China; 2212123021@stmail.ujs.edu.cn (F.F.); 2112323212@stmail.ujs.edu.cn (K.Z.); 2112223001@stmail.ujs.edu.cn (K.Z.); wangruijie@ujs.edu.cn (R.W.); xiajun@ujs.edu.cn (J.X.)

**Keywords:** bilayer graphene, grain boundaries, twist angles

## Abstract

Bilayer graphene exhibits intriguing physical and mechanical properties that are suitable for advanced electronic device applications. By introducing a new degree of freedom through interlayer twisting, exotic phenomena such as superconductivity can arise. However, in practical experiments, manual manipulation is often required to fabricate such a configuration and therefore, scaled production of magic angle bilayer graphene is challenging. In this work, we propose utilizing the grain boundaries and accompanying localized out-of-plane deformation in graphene to facilitate twisted bi-layer graphene formation. Based on molecular dynamics simulations, the structure folding process along the boundary line is examined where a lower energetic cost is found. Once stabilized, the folded bilayer structure shows twist angles that differ visibly from the conventional AA or AB stacking modes and can achieve twist angles close to the 1.1° magic angle. This observation suggests a potential novel strategy for synthesizing stable twisted bilayer graphene or other two dimensional van der Waals heterostructures with greater efficiency.

## 1. Introduction

Graphene has emerged as one of the most promising two-dimensional (2D) materials since it was first isolated in 2004 [1]. It has been demonstrated to host a wide range of extraordinary physical, mechanical, and optical properties that are suitable for advanced device applications ranging from high performance transistors, solid state lubricants, energy storage, and others [2,3,4]. Moreover, its properties have been shown to be layer dependent, for example, with increasing numbers of layers, its frictional characteristics show a monotonic variation trend and its band gaps can be opened in bilayer graphene due to the broken symmetry [5,6]. In naturally stacked graphene systems, the AB stacking configuration is the most stable state to form due to its lowest interlayer energy [7,8]. Recently, Cao et al. have shown that by introducing a slight twist in the bilayer graphene system at an angle of 1.1°, an anomalous correlation effect occurs and superconductivity can be observed [9]. This study opened up a new research area for twisting electronics in 2D van der Waals (vdW) materials [10,11,12,13,14,15]. However, the current method for achieving twist angles at specific values mostly relies on a repetitive trial-and-error process using tools such as atomic force microscopy with stringent sample preparation requirements. Moreover, such configurations can be easily disrupted during the transfer process or when exposed to external stimuli, such as high-temperature environments [16]. As a result, the fabrication of twisted bilayer vdW systems is challenging. It is therefore of great importance to investigate whether alternative approaches can be designed to achieve more robust twisted bilayer vdW materials at a lower expense.

Graphene is known to be mechanically strong when subjected to in-plane tension, with a Young’s Modulus of 1 TPa and a mechanical strength of 100 GPa [17]. But its out of plane bending stiffness is significantly lower compared to its in-plane strength [18]. This characteristic makes it easily bendable or foldable when subjected to vertical deflections. Experiments have often observed edge folding with a large area coverage occurring on exfoliated graphene layers [18,19,20]. These characteristics have motivated us to investigate an approach through graphene folding to achieve controlled misaligned stacking states, so that more efficient interlayer twist engineering can be achieved [21]. The key to this is the utilization of defective graphene, particularly when the defects form grain boundaries [22,23,24,25,26]. It is natural to expect that such a defect would exhibit distinct bending properties, which if properly designed could be used to guide the folding dynamics of graphene. Jayeeta Lahiri et al. have achieved a precise control over the defect structures found in graphene, providing crucial experimental evidence for studying the relationship between defect configurations and the properties of graphene. In recent years, great progress has been made in graphene folding techniques. By using the tip of an atomic force microscope (AFM) and STM to precisely pick-up and guide the placement of folded graphene, the accurate directional folding of graphene has been realized [19,27,28,29]. This progress provides an experimental foundation and theoretical support for the controlled morphological manipulation of graphene [23,30,31,32,33].

In this work, by using molecular dynamics simulations [34], we demonstrate the above scenario via the grain boundary guided folding of graphene. We found that by introducing grain boundaries into the lattice of graphene, the folding behavior of graphene occurs near the grain boundaries, called the folding line. We attribute this to the fact that the bending stiffness will be reduced once the folding line is close to the grain boundaries, and the asymmetric folding structure is realized due to the existence of the tilt angle at the folding line. Our results show that once the folding process is completed, the system’s energy reduces substantially owing to the vdW interaction between opposite layers, indicating the robustness of the deformed structure with respect to thermal or mechanical perturbation. We expect our results to guide future experimental efforts in designing twisted bilayer vdW structures.

## 2. Materials and Methods

**Figure 1:**
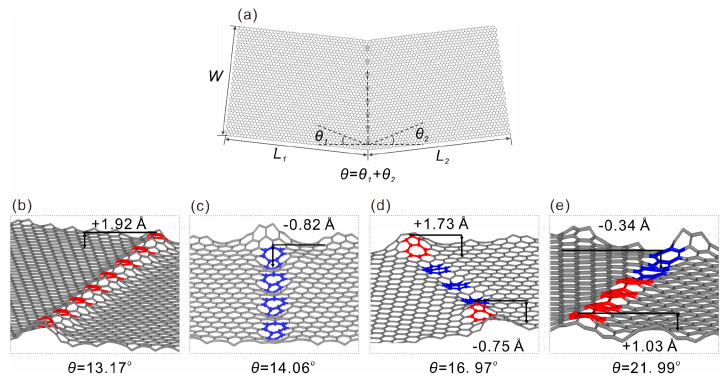
Type I folding line model constructed by discrete 7–5 defects along the central line of two graphene flakes. (**a**) geometric parameters of the model, two graphene with sizes *L_1_* × *W* and *L_2_* × *W* at slightly twisted angles of *θ_1_* and *θ_2_*, 7–5 type defects are introduced to connect the edge where two graphene flakes contact, forming a total grain boundary angle of *θ = θ_1_ + θ_2_*. (**b**–**e**) local structures of totally relaxed grain boundary with θ of 13.17°, 14.06°, 16.97°, and 21.99°. Out of plane deformation is clearly seen, the red colored atoms indicate upward deformation, while the blue atoms indicate downward deformation, the deformation amplitudes are also provided. The maximum out-of-plane deformation amplitudes for four types of graphene deformation are marked in (**b**–**e**).

**Figure 2:**
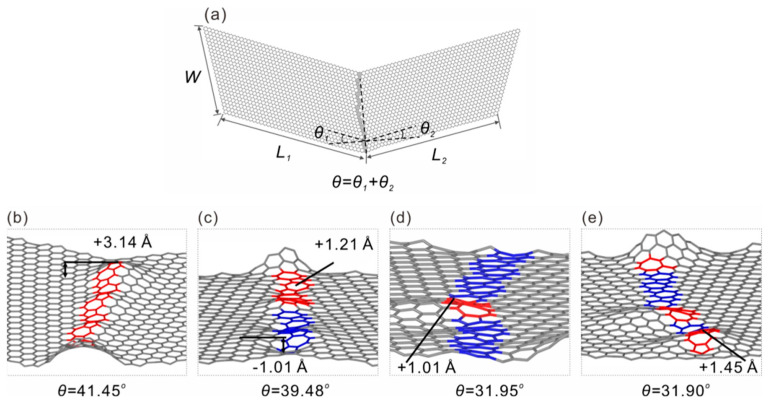
Type II folding line model with continuous sinuous type line defects in graphene, the definition of the geometrical parameters is the same as those defined earlier. (**a**) shows the overall structure, and (**b**–**e**) show local structures with apparent out of plane deformation. The maximum deformation amplitude values are marked in (**b**–**e**).

The molecular dynamics simulations were performed using the Large-scale Atomic/Molecular Massively Parallel Simulator (LAMMPS) package [35]. Grain boundaries in graphene were constructed by connecting two tilted graphene structures, with 5–7 polygon type defects discretely present along the boundary line separated by hexagonal rings (called the type I folding line), and the folding line is along a straight line, as depicted in Figure 1, where the overall tilt angle *θ*, is the sum of the two tilt angles *θ_1_* and *θ_2_*; this definition is the same as that provided by Zhang et al. [25]. In another type of structure (the type II folding line), the folding line runs through a grain boundary structure consisting of the continuously connected 7–5 topological defects (as shown in Figure 2) which forms a sinuous shape [30,36,37] and corresponds to the lowest total energy, according to Zhang et al. [25]. A silicon substrate is used in the simulation, but is not plotted in Figure 2 for a better visualization effect. The airebo interatomic potential model [38,39], which was initially developed for hydrocarbon modeling, was also used for carbon–carbon interactions. This model’s potential has been widely used and demonstrated to be accurate in simulating the mechanical behaviors of graphene and other carbon nanostructures [18,21,36]. The Stillinger–Weber potential model [40] was used to describe interactions between silicon atoms and the LJ potential was used to describe the interfacial vdW interaction [38,41]. Before the folding simulation, an energy minimization using the conjugate gradient energy minimization method was used to obtain optimized structures [34]. The folding simulation was realized by pulling up the right end of the graphene then bending it towards the left end, the structure was then subjected to relaxation before obtaining the final folded structure. The Nose–Hoover thermostat was used throughout the simulations to maintain a constant temperature of 1 K in order to exclude thermal fluctuation effects [42]. A timestep of 1 fs was chosen for the integration of the trajectories of atoms. Visualization and structural analysis were performed using the Open Visualization Tool (OVITO) [43].

## 3. Results

Shown in Figure 1a is a representative atomic model of the type I graphene folding line, where two graphene monolayers with *L_1_* × *W* and *L_2_* × *W* in size are tilted at angles of *θ_1_* and *θ_2_*; the overall tilt angle is *θ = θ_1_ + θ_2_.* The 5 and 7 membered rings are used to construct the grain boundaries and with an increased tilt angle, it is straightforward to see that the number of 5–7 defects increases, consistent with those reported in the literature [22,30]. Interestingly, with the introduction of defects to the folding line, the graphene structure shows notable out of plane deformation at the defect sites, as can be seen in Figure 1b–e. Although there is no notable monotonic trend of deformation amplitude with increasing defect concentrations (i.e., the grain angle *θ*), and the deformation direction is random as well, this behavior is, however, encouraging for the folding process. One may expect such defects to lower the bending stiffness as compared to pristine graphene, and moreover, the bending energy will be naturally reduced. For the type II folding line shown in Figure 2, similar behavior was also observed. The major difference here was that the folding line was not a straight line as it costed a higher amount of energy; this finding is consistent with the previous prediction of sinuous grain boundaries made by Zhang et al. [25]. Similar to those observed in the 7–5 grain boundaries, the sinuous defect sites also exhibited out-of-plane deformation in the material. Most of the deformation amplitudes were around 1~2 Å, which shows that the deformation amplitude between the two folding line models was similar.

**Figure 3:**
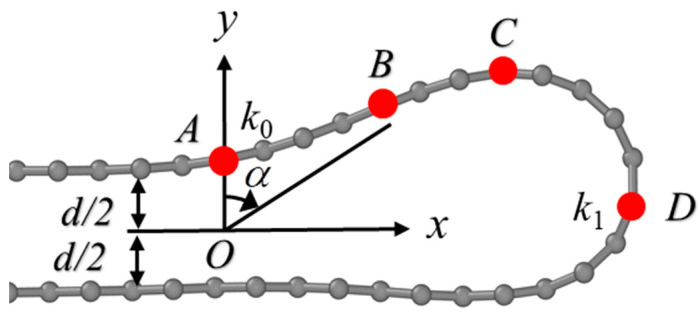
Geometrical model used to calculate bending stiffness of folded graphene. The left region to atom A (marked in red) is the flat region with zero curvature, the regions from A to B (with curature of *k_0_*), B to C, and C to D (with curvature of *k_1_*) represent typical curved geometries that are used in bending stiffness calculations. The definition of angle α in Equation (2) is also given.

We now proceed to calculate the bending stiffness of the defective graphene. It is interesting to note that during the folding simulations, the ultimate stabilized bilayer structures had folding lines located exactly at the grain boundary, as shown in the structure illustrated in Figure 3, hinting that folding at the grain boundary is likely to cost the lowest in terms of bending energy, as will be demonstrated below. The total energy of the bent graphene in Figure 3 can be expressed as the following:

(1) $$U_{\mathrm{total}}=U_{b}-U_{\mathrm{vdw}}$$

where U_vdW_ is the van der Waals energy between opposite layers and U_b_ is the bending energy which is contributed by the folded section at the highly curved region. Following the analysis provided by Zhang et al. [44], U_b_ can be written as the following:

(2) $$U_{b}=\mathrm{EI}\left[2\int_{0}^{\sin^{-1}\frac{k_{0}^{2}}{k_{1}^{2}-k_{0}^{2}}}\sqrt{k_{0}^{2}-(k_{1}^{2}-k_{0}^{2})\sin\alpha }\;d\alpha \right.\left.+\int_{0}^{\frac{\pi }{2}}\sqrt{k_{0}^{2}+(k_{1}^{2}-k_{0}^{2})\sin\alpha }\;d\alpha \right]$$

where *k*_1_ is the curvature at the right end point of the folded graphene, *k*_0_ is the curvature at the joint position of the flat graphene and curved segment, and α is the angle between y axis and the vector of a carbon atom with respect to the center of the coordinate, as plotted in Figure 3. Although it is challenging to determine how far from the folding line that the stabilization effect can be perceptible in the plane of graphene sheets, the current model gives a reasonable estimation of the total energy and bending energy [44]. The bending stiffness can be obtained by taking the second partial derivative of the above equation to *k*_1_.

**Figure 4:**
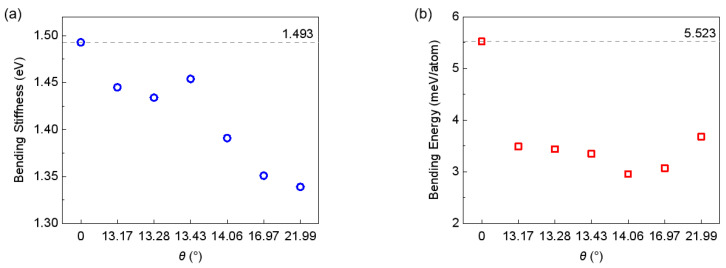
Bending stiffness (**a**) and bending energy (**b**) of type I graphene boundaries. The dashed lines indicate corresponding values of defect free graphene.

**Figure 5:**
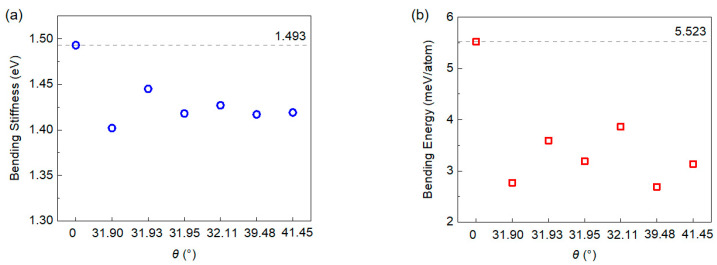
Bending stiffness (**a**) and bending energy (**b**) of type II graphene boundaries. The dashed lines indicate corresponding values of defect free graphene.

The obtained bending stiffness for both the type I folding line and the type II folding line are summarized, as shown in Figure 4 and Figure 5. Our calculation of the bending stiffness of pristine graphene is 1.493 eV, which is close to the value reported by Wei et al. [18]. When introducing grain boundaries into graphene and bending it along the grain boundary, it is not surprising to see that both the bending stiffness and bending energy of the graphene is substantially reduced. With the bending stiffness reducing from 1.493 eV to about 1.45 eV at a tilt angle of 13.17°~13.43°, upon a further increase of tilt angle up to 21.99°, the bending stiffness continuously decreases to about 1.33 eV. Meanwhile, the bending energy is reduced from 5.523 meV/atom for pristine graphene to about 3.5 meV/atom for graphene grain boundaries, and interestingly, the bending energy does not show strong correlation with the tilt angle of the grain boundary. However, the overall results are supportive of the above mentioned idea that the existence of the grain boundary facilitates the folding of the graphene monolayer. Previous experiments [19,45] showed that for pristine graphene, folding edges are primarily of an armchair or zigzag type, thus the stacking order in the folded bilayer graphene is mainly AA or AB. According to our simulations, because folding along grain boundaries reduces the overall energy required for bending, it is natural to expect that using grain boundaries to guide the folding process—with the introduction of a tilt angle—the resultant stacking order could be of the twisted bilayer graphene type.

**Figure 6:**
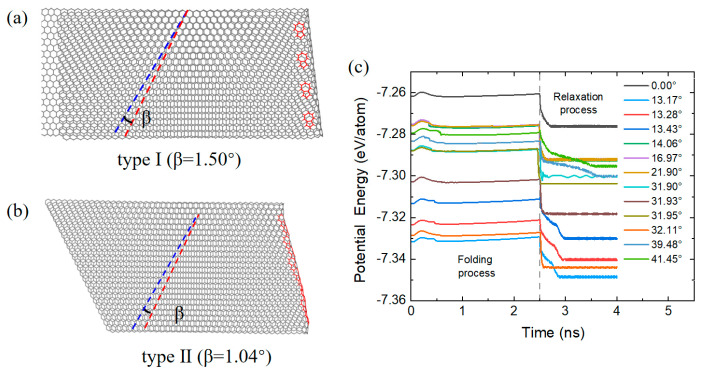
Representative folded bilayer graphene structures with two types of folding line. A twist angle of *β* = 1.50° (**a**) is achieved in one of the type I models and *β* = 1.04° a twist angle is achieved in (**b**) for the type II model. Potential energy variation during the folding and relaxation process is shown in (**c**). The tilt angles range from 0 to 41.45° as shown in (**c**).

The above scenario was demonstrated in our simulations as shown in Figure 6; here the graphene structures of various tilt angles ranging from 0° to 41.45° were investigated to examine their folding dynamics. The bending process is achieved via continuously applying out-of-plane deformation to the right side of the graphene grain boundary. Once the bending angle reaches 160° at 2.5 ns, the structure is allowed to relax, owing to the vdW attractive interaction between opposite layers, the structure automatically evolves towards the 180° bending angle. In other words, it is completely folded. From the potential energy plot, it can be seen that for all the structures, the manual bending process raises the potential energy slightly, owing to the increased curvature. Once the structural relaxation started, the vdW interaction between opposite layers played a key role in rapid decreasing the potential energy of the folded structure; the magnitude of the energy drop was around 2.8~3.6 meV/atom. Interestingly, after folding, the bending edges (or folding lines) were all found to be around the grain boundaries, consistent with the above analysis that out of plane deformation facilitates the bending process. Moreover, it was observed that all the resultant bilayer structures exhibited a slight twist between neighboring layers. The twist angles are found to be ranging from 0.12° to 1.74°. This result hints a possible strategy to fabricate twisted bilayer graphene with small twist angles. For example, as shown in Figure 6a, a type I graphene structure with a tilt angle of 14.06° is folded along the straight 7–5 folding line, and after folding, the resultant twist angle is found to be *β* = 1.5°. For the type II graphene folding line with a tilt angle of 32.11°, the simulation yielded a twist angle of *β* =1.04°, which is close to the magic angle for a superconductive state. What was unexpected is that our simulation did not yield any correlation between the ground boundary angle and the ultimate twist angle. Additional tests could be performed to find ways to construct bilayer graphene with larger twist angles. We also note that the types of defects considered in this study does not include all possible scenarios, such as point defects or octagon defects. Future works could be performed to examine how these impact folding behavior.

## 4. Conclusions

In conclusion, we have proposed a strategy for the fabrication of small twist angle bilayer graphene through the assistance of grain boundary structures. Our molecular dynamics simulations show that by introducing topological defects that form grain boundary, localized out-of-plane deformation occurs at the defect sites. When bent along these deformed positions, both the bending stiffness and bending energy are reduced compared to bending along the defect-free lattice structure. As a result, the folded bilayer structures are found to stabilize in conformations where the fold lines align with the grain boundaries. Detailed measurements show that after folding, the bilayer structures do not show regular AA or AB stacking states; instead, slight twists between neighboring layers are found, with some of the structures exhibiting a magic angle stacking character. These results demonstrate a novel synthesis pathway for twisted bilayer graphene with a tailored structure, which could stimulate experimental efforts to develop scalable fabrication methods. However, precise control over interlayer twist angles remains a critical challenge that requires further systematic investigation.

## Data Availability

The study data are contained within the article.
